# The association of serum levels of vitamin D with leucocyte telomere length, as a marker of biological aging: A meta-analysis

**DOI:** 10.1097/MD.0000000000044487

**Published:** 2026-02-06

**Authors:** Jie Shen, Lin Wang, Jiyuan Liu, Zhichao Fan, Guanghui Li

**Affiliations:** a Hebi Vocational and Technical College, Hebi, China; b General Surgery Ward 2, Hebi People’s Hospital, Hebi, China; c Medical Affairs Management Department of People’s Hospital of Zhengzhou, Zhengzhou, China.

**Keywords:** 25(OH)D, meta-analysis, telomere length, vitamin D

## Abstract

**Background:**

Short telomere length (TL) has been associated with chronic diseases and reduced lifespan. Vitamin D may help preserve telomeres through its anti-inflammatory effects; however, the relationship between serum 25-hydroxyvitamin D (25(OH)D) levels and TL remains inconclusive. This meta-analysis was conducted to evaluate the association between circulating 25(OH)D and leukocyte TL (LTL).

**Methods:**

A comprehensive literature search was performed across PubMed, Scopus, Google Scholar, ClinicalTrials.gov, and Cochrane Library to identify relevant studies published up to February 2025. Standardized β coefficients with 95% confidence intervals were applied as the effect size metric to evaluate the associations using a random effect model.

**Results:**

A total of 21 studies comprising 185,191 participants were analyzed. The overall results demonstrated a positive association between serum 25(OH)D levels and LTL (β = 0.04, 95% CI = 0.02–0.06), with remarkable heterogeneity across studies (I²= 89.1%, *P* ≤.001). This association was supported in adults (β = 0.04, 95% CI = 0.03–0.06), women (β = 0.05, 95% CI = 0.01–0.08), individuals with vitamin D deficiency (β = 0.22, 95% CI = 0.01–0.43), and studies that adjusted for covariates (β = 0.05, 95% CI = 0.01–0.08). No significant associations were found in men, participants with serum 25(OH)D levels ≥ 30 ng/mL, children, or studies without covariate adjustments. The relationships were not influenced by the method of TL assessment, body mass index, smoking status, and sample size.

**Conclusion:**

Serum 25(OH)D levels showed a positive correlation with LTL in women, adults, and individuals with vitamin D deficiency.

## 1. Introduction

Telomeres, which are nucleoprotein caps composed of repetitive nucleotide sequences and telomeric proteins located at the chromosomal ends, play a crucial role in maintaining genomic stability and cellular function.^[1]^ Telomere length (TL) naturally shortens during cell division and aging and shortened TL is known as a main biomarker of aging.^[2]^ In humans, TL declines at a rate of approximately 24.8 to 27.7 base pairs annually.^[3]^ TL varies among individuals of the same chronological age since it is influenced by a blend of intrinsic factors, such as genetics, and extrinsic factors, including environmental exposures, lifestyle, and diet.^[4,5]^ Accelerated telomere attrition has been linked to various age-related diseases, including cardiovascular disease, diabetes, cancer, osteosarcopenia, neurodegenerative disorders, and to reduced lifespan.^[6]^ Identifying factors that influence telomere dynamics is essential for understanding mechanisms of aging and exploring potential interventions to promote healthy aging.

Vitamin D, a fat-soluble vitamin known for its role in calcium homeostasis and bone health, has gained significant attention in research due to its broader implications in human health. Beyond its classical functions, vitamin D has been implicated in modulating inflammation, immune function, oxidative stress, apoptosis, and cell differentiation, mechanisms known to contribute to telomere shortening.^[7]^ Vitamin D insufficiency has been linked to age-related diseases.^[8]^ Accordingly, serum levels of 25-hydroxyvitamin D (25(OH)D), the primary marker of vitamin D status, have been hypothesized to be associated with leucocyte TL (LTL). The presence of vitamin D receptors in leukocytes indicates that vitamin D may potentially impact LTL. While individual studies have investigated the potential link between serum 25(OH)D and LTL, their findings have been inconsistent. Some studies have revealed a direct association between serum 25(OH)D and LTL. Richards et al.^[9]^ and Liu et al.^[10]^ found a positive correlation between serum 25(OH)D levels and LTL in women.^[11]^ However, a cross-sectional study involving 2483 men aged 40 to 75 years failed to observe a similar positive association between 25(OH)D and LTL.^[2]^ Williams et al.^[12]^ also indicated that vitamin D status may not be a significant factor influencing LTL. These discrepancies highlight the need for a comprehensive synthesis of the available evidence to clarify the nature of this association.

The potential sources of heterogeneity across the studies may be attributed to the differences in key factors such as studied population, age, gender, vitamin D status, and methodological differences among studies. To address this gap, we conducted a meta-analysis of observational studies to examine the relationship between serum 25(OH)D levels and LTL.

## 2. Materials and methods

This study was conducted and reported according to the guidelines set by the Preferred Reporting Items for Systematic Reviews and Meta-Analyses (PRISMA) Statement.^[13]^ Since this study does not include the recruitment of patients or the collection of personal information, ethical approval and patient consent were not required for this type of study.

### 2.1. Literature search

A systematic search was conducted on PubMed, Scopus, Google Scholar, ClinicalTrials.gov, and Cochrane Library for relevant articles published prior to February 2025 to assess the relationship between serum 25(OH)D and TL. The following search strategy was applied: (“Vitamin D”[Mesh] OR “Vitamin D Deficiency”[Mesh] OR Vitamin D[Title/Abstract] OR 25-hydroxy-vitamin D[Title/Abstract] OR 25OHD[Title/Abstract] OR calcitriol[Title/Abstract] OR “25-hydroxy vitamin D”[Title/Abstract] OR “25 hydroxyvitamin D”[Title/Abstract] OR 25(OH)D[Title/Abstract] OR Calciferol[Title/Abstract] OR colecalciferol[Title/Abstract] OR vitamin D 3 [Title/Abstract]) AND (“Telomere”[Mesh] OR “Telomere Shortening”[Mesh] OR telomere[Title/Abstract] OR telomeres[Title/Abstract]). No restrictions were placed on publication dates, and only English-language articles were considered for inclusion. Additionally, the reference lists of included studies and relevant reviews were manually examined to identify potential additional literature. The search was conducted by 2 investigators, and duplicates were eliminated. Both investigators independently reviewed article titles, abstracts, and full texts, and any disagreements were resolved through consensus.

### 2.2. Selection criteria

Articles were included if they met the following criteria: were observational in design, examined the relationship between serum 25(OH)D and TL, used leucocytes as the DNA source, and provided a standardized beta (β) coefficient with 95% confidence intervals for the association, or presented enough information to calculate these values. Studies with insufficient information, case reports, reviews, comments, editorials, and studies on supplemental or dietary vitamin D use, and studies with unrelated exposure/outcome were excluded. Two investigators independently evaluated the eligibility of studies, and any discrepancies resolved through involvement of a third author.

### 2.3. Data extraction and quality assessment

The following details were obtained from the included publications: the first author, publication year, country, gender of subjects, frequency of smokers, total sample size, study design, the method used to measure TL, mean of age, serum 25(OH)D level, body mass index (BMI), the β coefficient with confidence intervals for the association between serum 25(OH) and TL, and covariates adjusted for in the analyses. The quality of studies was evaluated using a modified version of the Newcastle-Ottawa Scale (NOS). The NOS has a maximum score of 9, evaluating aspects such as selection, comparability, and outcome. The studies were categorized based on their scores: those with scores of 0 to 3 were deemed low quality, scores of 4 to 6 were considered medium quality, and scores of 7 to 9 were regarded as high quality.^[14]^

### 2.4. Statistical analysis

To examine the relationship between serum 25(OH)D and TL, the standardized β coefficient with a 95% CI was used as the effect size. The Cochran-Q test and I-squared (I^2^) statistics were applied to evaluate heterogeneity across the publications. Heterogeneity was indicated if the I^2^ value exceeded 50% or the Cochran-Q *P*-value was <.1.^[15,16]^ A random-effects model with DerSimonian and Laird was applied to pool the data.^[17]^ The prediction interval was calculated to estimate the range in which the true effect size of a future study is expected to fall, taking into account both the overall effect and between-study heterogeneity.^[18]^ Subgroup analyses were conducted by population (adults [aged ≥ 18 years] versus children [aged <18 years]), sample size (≥1000 vs <1000 participants), gender, BMI category (≥25 vs <25 kg/m2), vitamin D status (≥30 vs <30 ng/mL), method of TL assessment (qPCR vs Southern blot), and covariate adjustment (yes vs no) to identify potential sources of heterogeneity. Meta-regression analyses were further performed to investigate heterogeneity based on factors such as sample size, BMI, smoking status, study quality, and sex ratio (male %). Sensitivity analyses were conducted by removing individual studies one by one to evaluate the impact of each study on the overall effect size. The Egger regression test was used to assess publication bias, with *P* <.1 considered significant.^[19,20]^ If publication bias was identified, a trim-and-fill analysis was applied to adjust the results for publication bias.^[21]^ STATA 13.0 software was employed for the meta-analysis.

## 3. Results

### 3.1. Study characteristics

**Figure 1.**
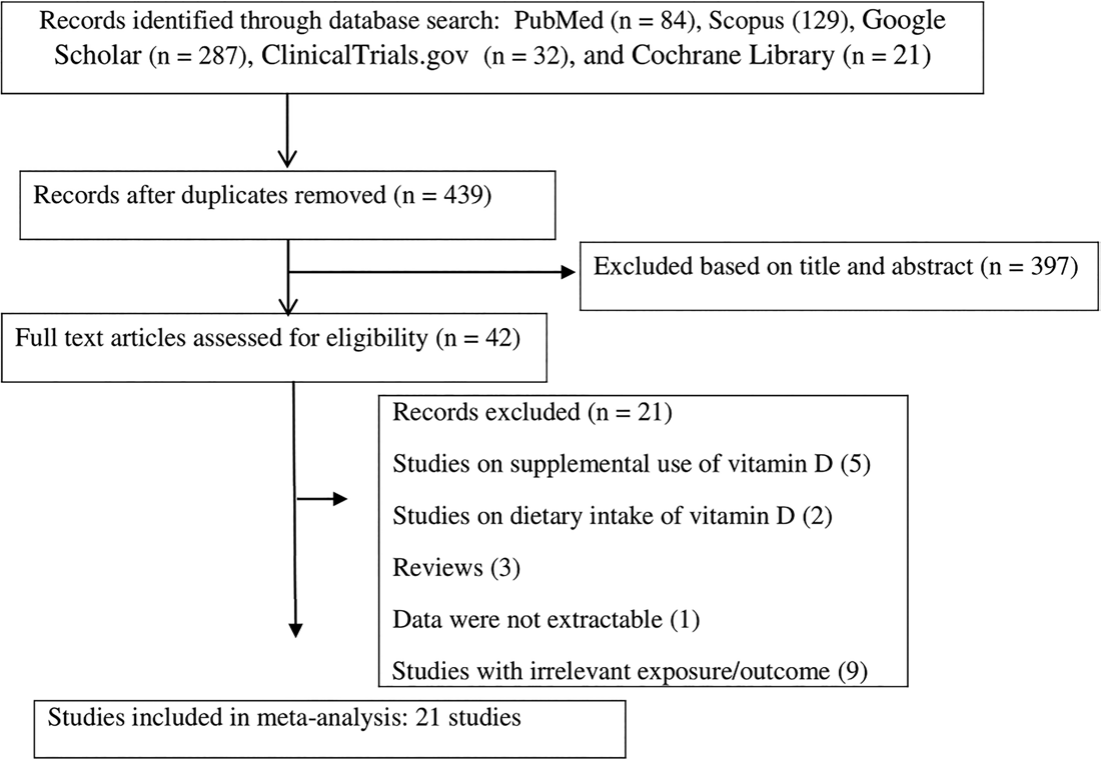
Flow diagram of the study.

A systematic literature review identified 553 publications, from which 114 duplicates were removed. Following title and abstract evaluation, 397 irrelevant articles were excluded, resulting in 42 potentially relevant studies that underwent full-text evaluation. After further screening, 21 additional studies were excluded due to reasons like being review articles, focusing on supplemental use of vitamin D, having irrelevant exposures/outcomes, or lacking sufficient data. Ultimately, 21 studies^[1,2,6,9–12,22–35]^ published between 2007 and 2023, involving a total of 185,191 participants, were included in the meta-analysis. The study selection process is illustrated in Figure 1. All included studies employed a cross-sectional design. Among the included studies, 11 reported results for a combined population of both genders, 6 studies separately analyzed results for male and female subgroups,^[1,11,24,26,29,32]^ 3 studies focused solely on women,^[9,10,25]^ and 1 study examined only men,^[2]^ yielding a total of 27 effect sizes. The mean serum 25(OH)D levels in these studies ranged from 19.7 ± 6.74 ng/mL to 88.5 ± 32.0 ng/mL. Participant ages spanned from 8.8 ± 1.7 years to 64.3 ± 7.5 years. Most studies (19 studies) focused on adults, while 2 focused on children.^[1,23]^ For LTL assessment, 19 studies used quantitative polymerase chain reaction (qPCR)^[1,2,6,10–12,22–24,26–35]^ and 2 used the Southern blot method.^[9,25]^ The majority of the studies adjusted for potential covariates, although 4 presented crude results without controlling for covariates.^[22,30,33,35]^ The methodological quality of the included studies, assessed using NOS, ranged from medium to high, with scores between 4 and 9. The characteristics of the included publications are detailed in Table 1.

**Table 1:** Characteristics of studies.

Reference	Year	Country	Male (%)	N of participants	Mean serum 25(OH)D (ng/mL)	Age (mean ± sd)	Mean BMI (kg/m^2^)	Smokers (%)	TL assessment	Adjustment
Kuo	2023	USA	49.70	148,321	51.34 *± *20.65	64.13 *± *2.85	24.9 ± 8.84	7.9	qPCR	Age, sex, ethnicity, Townsend deprivation index, education, whole body fat mass, smoking status, alcohol intake, and physical activity, and serum calcium
Al-Daghri	2022	Saudi Arabia	48.30	775	44.2 *± 17.1*	55.9 ± 8.2	30.9 ± 6.0	NR	qPCR	Not adjusted
Beilfuss	2016	USA	48.20	4260	62.6 ± 6 1.2	48.6 ± 1.0	28.71 ± 0.32	NR	qPCR	Age, sex, race/ethnicity, BMI, total energy and sugar intakes, calcium intake, socioeconomic status, consumption of milk and dietary supplements, and physical activity
Normando	2019	Brazil	48.5	464	48.3 ± 20.4	51.4	NR	36.2	qPCR	Age, sex, marital status, equivalent per capita household income, educational attainment, physical activity, smoking status, diagnostic of chronic disease, skin type, month of blood collection, sun exposure index, visceral fat mass, and CRP
RIBERO	2016	UK	0	3501	75 ± 73.75	46.5 ± 15.24	NR	NR	Southern blot	Season, height, weight and age
Mazidi	2016	USA	47	4347	23.3 ± 8.8	42.7	27.5 ± 6.1	20.4	qPCR	Age, race, marital status, education, CRP, smoking, BMI and physical activity
Bhatt	2021	India	59	300	29.8 ± 9.4	42.6 ± 8.6	26.8 ± 6.9	NR	qPCR	Age and sex
Akash	2021	India	69	90	25.43 ± 10.69	58.29 ± 13.87	24.04 ± 2.03	13.3	qPCR	Not adjusted
Schottker	2018	Germany	45.10	3564	51.1 ± 24.6	62.1 ± 6.6	NR	NR	qPCR	Age, sex, season, BMI, education, smoking, physical activity, history of cancer, and history of cardiovascular disease, and study batch
Liu	2013	USA	0	4604	NR	59.3 ± 6.3	25.7 ± 4.9	NR	qPCR	Study batch, age, smoking status, BMI, and physical activity
Williams	2015	UK	48.20	5096	50.6 ± 14.9	31.1 ± 0.35	24.0 ± 2.74	63.2	qPCR	Season, age, sex, 25(OH)D batch, BMI, socioeconomic position, physical activity, diet, smoking, alcohol intake, use of oral contraceptives (women only), and CRP
Milne	2015	Australia	50	450	88.5 ± 32.0	3–9 years	16.41 ± 2.6	NR	qPCR	Age, sex, month of blood collection
Dudinskaya	2023	*Russia*	33.30	305	19.7 ± 6.74	48.82 ± 13.87	24.2	NR	qPCR	Not adjusted
Wijayabahu	2022	USA	37	402	NR	56.6 ± 7.6	NR	21.1	qPCR	Age, sex, race, study site, WHR, physical activity, and number of pain sites
Julin	2017	USA	100	2483	33.3 ± 12.3	64.3 ± 7.5	25.6 ± 3.1	53	qPCR	Batch of vitamin D assay, age, smoking, BMI, physical activity, alcohol consumption, season of blood collection
Richards	2007	UK	0	2160	78.9 ± 41.3	49.4 ± 12.9	25.3 ± 4.5	18.4	Southern blot	Age, season of vitamin D measurement, menopausal status, use of hormone replacement therapy, and physical activity
Bussa	2022	Colombia	47.40	447	73.6 ± 22.8	8.8 ± 1.7	NR	NR	qPCR	Age, height, BMI, and socioeconomic status
Hoffecker	2013	USA	NR	59	41.61 ± 11.18	39.86 + 11.57	NR	NR	qPCR	Not adjusted
Liu	2023	USA	38.3	1154	NR	48–93	NR	9	qPCR	Age, season, race
Vetter	2020	Germany	49.1	1634	NR	48.7	25.05 ± 4.1	NR	qPCR	Age, sex, alcohol intake, packyears, morbidity index, BMI, season of blood draw
Hakeem	2021	UK	39.2	775	NR	NR	NR	59.6	qPCR	Smoking, alcohol intake, BMI, physical activity, and HbA1c%.

BMI = body mass index, CRP = C-reactive protein, HbA1c = hemoglobin A1c, NR = not reported, qPCR = quantitative polymerase chain reaction, TL = telomere length, WHR = waist–hip ratio.

### 3.2. Quantitative analysis

**Figure 2.**
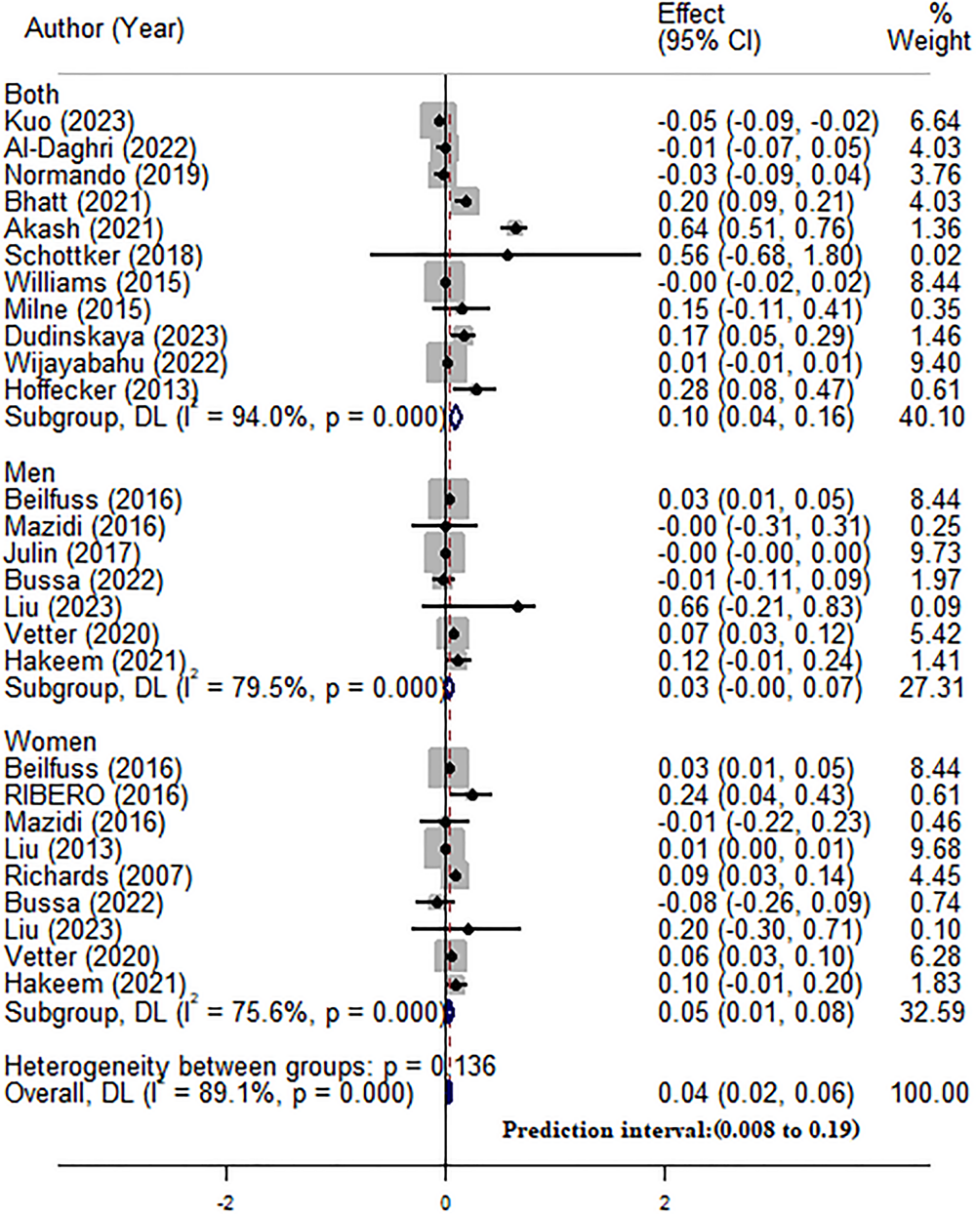
Overall and subgroup analysis by the gender of participants for the association between serum vitamin D and TL. TL = telomere length.

The pooled analysis of all available studies found a positive correlation between serum 25(OH)D levels and LTL (β = 0.04, 95% CI = 0.02–0.06; Fig. 2), although significant heterogeneity was observed among the studies (I2 = 89.1%, *P* ≤ .001). Subgroup analyses revealed significant associations in women (β = 0.05, 95% CI = 0.01–0.08), participants with vitamin D deficiency (β = 0.22, 95% CI = 0.01–0.43), adults (β = 0.04, 95% CI = 0.03–0.06), and studies adjusting for covariates (β = 0.05, 95% CI = 0.01–0.08). Conversely, no significant relationships were observed in men, participants with serum 25(OH)D levels ≥ 30 ng/mL, children, or studies not adjusting for covariates. The association between serum 25(OH)D and LTL was unaffected by the LTL assessment method, BMI, or sample size (Table 2).

**Table 2:** Overall and subgroup analysis for the association between telomere length and serum vitamin D (25(OH)D) level.

Prediction interval	Test of heterogeneity		Test of association		Studies (effect sizes)	Subgroups	Subgroup variable
	*P*	*I*^2^ (%)	95% CI	β			
0.008–0.19	<.001	89.1	0.02 to 0.06	0.04	21 (27)	Overall	Overall
−0.04 to 0.38	<.001	79.5	−0.001 to 0.07	0.03	7 (7)	Men	Gender
0.007–0.31	<.001	75.6	0.01 to 0.08	0.05	9 (9)	Women	
0.007–0.99	<.001	94.0	0.04 to 0.16	0.10	11 (11)	Both	
0.005 to 0.06	<.001	80.3	0.01 to 0.03	0.02	11 (15)	≥ 1000 participants	Sample size
0.009 to 0.98	<.001	92.9	0.04 to 0.20	0.12	10 (12)	< 1000 participants	
0.004 to 0.08	<.001	82.2	0.01 to 0.04	0.02	17 (23)	Yes	Adjustment for covariates
-0.10 to 0.91	.12	45.8	−0.04 to 0.57	0.27	4 (4)	No	
0.01 to 0.14	<.001	90.3	0.03 to 0.06	0.04	19 (24)	Adults	Population
-0.13 to 0.15	.35	3.3	−0.09 to 0.08	−0.01	2 (3)	Children	
-0.09 to 0.16	<.001	75.7	−0.01 to 0.03	0.01	12 (14)	≥ 30 ng/mL	Serum 25(OH)D
0.001 to 0.99	<.001	91.9	0.01 to 0.43	0.22	4 (5)	< 30 ng/mL	
0.005 to 0.15	<.001	77.5	0.01 to 0.05	0.03	5 (8)	NR	
0.006 to 0.13	<.001	88.7	0.02 to 0.05	0.03	8 (10)	≥ 25 Kg/m^2^	BMI
0.001 to 0.99	<.001	96.6	0.03 to 0.20	0.17	5 (5)	< 25 Kg/m^2^	
0.01 to 0.33	<.001	68.7	0.01 to 0.10	0.06	8 (12)	NR	
0.009 to 0.16	<.001	89.3	0.02 to 0.05	0.04	19 (25)	qPCR	Method of TL assessment
0.001 to 0.41	.14	52.5	0.01 to 0.27	0.13	2 (2)	Southern blot	

BMI = body mass index, qPCR = quantitative polymerase chain reaction, TL = telomere length.

### 3.3. Sensitivity analysis

**Figure 3.**
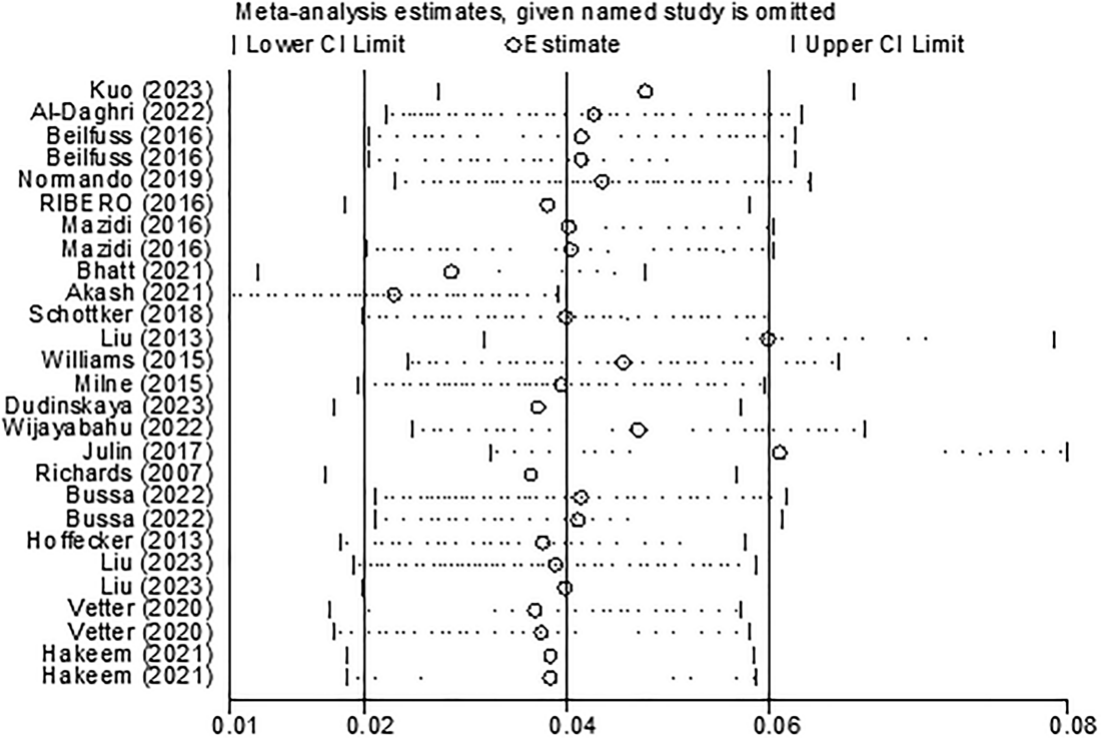
Sensitivity analyses by excluding 1 study at a time.

A sensitivity analysis showed that the pooled effect size remained relatively consistent after sequentially removing individual studies and re-analyzing the remaining effect sizes, indicating reliability of the findings (Fig. 3).

### 3.4. Meta-regression analysis and publication bias

**Figure 4.**
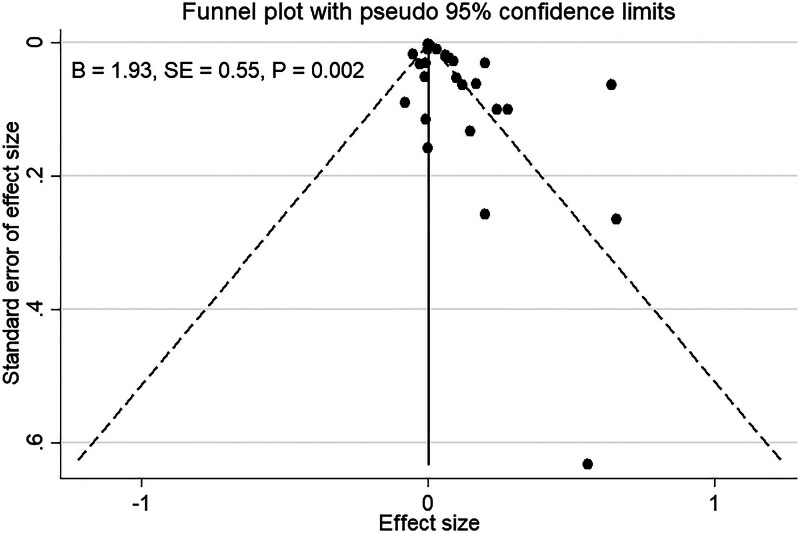
Funnel plot for publication bias.

The Egger regression test revealed significant evidence of publication bias (Fig. 4). However, when adjusting for this bias using a trim-and-fill analysis, the corrected pooled effect size remained similar (corrected β = 0.03, 95% CI = 0.01–0.06), suggesting that publication bias had a minimal impact on the overall findings. Additionally, meta-regression analyses showed that quality score of studies, sample size, BMI, and smoking status did not significantly influence the associations (all *P*-values ≥.10).

## 4. Discussion

In this study, we examined the association between 25(OH)D levels and LTL. We found a positive relationship between serum 25(OH)D levels and LTL in women, and those with vitamin D deficiency, while no significant associations were found in men, children, or individuals with sufficient vitamin D levels.

Men generally have shorter telomeres than women, which could explain why there are sex differences in the relationship between vitamin D levels and TL.^[2,36]^ In line with our findings, the sex-specific association between 25(OH)D levels and LTL has been suggested in some previous studies.^[2]^ Several biological factors might contribute to these differences. Hormonal variations, for instance, could affect how vitamin D influences telomeres.^[37]^ Estrogen, unlike testosterone, can stimulate telomerase production and reduce reactive oxygen species, potentially protecting telomeres.^[38]^ Additionally, since males have an XY chromosome configuration, any defects in genes responsible for telomere maintenance on the unpaired X chromosome could lead to shorter telomeres, as there is no second X chromosome to compensate for such flaws.^[39]^

Consistent with our results, previous studies has indicated that the relationship between vitamin D and LTL may vary at different stages of the life cycle.^[1]^ The observed positive association between serum 25(OH)D levels and LTL in adults, but not in children, can be justified by several factors. Telomeres naturally shorten with age due to cumulative cellular replication and exposure to oxidative stress. Adults may experience greater telomere attrition compared to children due to a higher long-term exposure to environmental factors (e.g., geographic latitude, sun exposure, nutrition, physical activity) over the course of their entire life span, making the protective effects of vitamin D more pronounced in adults.^[1]^ Vitamin D deficiency is more common in adults due to lifestyle factors such as reduced outdoor activity or aging-related changes in vitamin D metabolism.^[40]^ In populations with deficiency, anti-inflammatory and antioxidant effects of vitamin D may help preserve TL, while children with sufficient vitamin D levels may not exhibit the same correlation. Vitamin D has been shown to influence telomerase activity, reduce oxidative stress, and modulate inflammation, all of which contribute to telomere maintenance.^[32]^ These mechanisms might be more relevant in adults who are at higher risk for chronic inflammation and oxidative damage compared to children. Children are in a phase of rapid growth and cellular proliferation, during which telomere dynamics may differ from those of adults.^[41]^ The role of vitamin D in telomere maintenance might be less evident during this developmental stage. These findings suggest that the protective effects of vitamin D on telomeres may become more significant with age due to increased exposure to factors that accelerate telomere shortening. Further research is needed to explore these age-specific differences in greater detail.

Optimal vitamin D levels could protect against TL shortening through various mechanisms, including its anti-inflammatory effects, reduction of oxidative stress, improvement of telomerase activity, regulation of cellular differentiation, proliferation, and apoptosis, as well as promotion of genomic stability.^[7,32]^ Vitamin D reduces inflammation, which is a major contributor to telomere shortening.^[42]^ Chronic inflammation leads to oxidative stress and cellular damage, accelerating telomere attrition. Besides, reactive oxygen species (ROS) can damage the guanine-rich regions at the ends of chromosomes, known as telomeres, and stimulate cell division, ultimately accelerating telomere shortening.^[43]^ The anti-inflammatory and antioxidant properties of vitamin D may help preserve TL by mitigating these effects.^[32]^ Vitamin D may also influence the activity of telomerase, an enzyme responsible for maintaining TL by adding nucleotide sequences to chromosome ends.^[7,43]^ Low levels of telomerase activity result in shorter telomeres, signaling cell senescence and apoptosis. Vitamin D supplementation has been linked to improved telomerase function, which may prevent excessive telomere shortening.^[42]^ Furthermore, vitamin D regulates vital cellular processes such as differentiation, proliferation, and apoptosis.^[44]^ These functions are crucial for maintaining healthy telomeres and preventing premature cellular aging. Vitamin D also contributes to genomic integrity by reducing DNA damage and supporting repair mechanisms necessary for stable telomeres.^[45]^ These mechanisms highlight the multifaceted role of vitamin D in cellular health and its potential protective effects against age-related diseases through the maintenance of TL.

The findings of the current study have several clinical utilities. The positive association between 25(OH)D levels and LTL in women, adults, and individuals with vitamin D deficiency suggests that targeted vitamin D supplementation could be beneficial for these groups to reduce telomere shortening. The lack of association in men highlights the need for gender-specific health interventions. Understanding these gender differences can guide more effective public health strategies. Regular monitoring of vitamin D levels, especially in populations prone to deficiency (e.g., obese and postmenopausal women),^[46,47]^ could help identify individuals who might benefit from supplementation to maintain LTL. Since longer telomeres are associated with lower risks of chronic diseases,^[6]^ maintaining adequate vitamin D levels could be part of broader preventive strategies against age-related conditions. Conducting longitudinal studies to assess how changes in vitamin D levels over time affect LTL would provide valuable insights into causality and temporal relationships. Moreover, randomized controlled trials examining the effects of vitamin D supplementation on LTL in specific populations (e.g., vitamin D-deficient women) would be crucial for establishing causality and clinical utility.

This meta-analysis is the first to investigate the relationship between serum 25(OH)D levels LTL. The strengths of the present study include its large sample size, comprehensive subgroup analyses, and meta-regression analyses that account for various demographic and lifestyle factors influencing vitamin D and LTL, facilitating to identify sources of heterogeneity. Stratified analysis by BMI was also conducted, as obese and overweight individuals often have lower vitamin D levels. However, there are several limitations to consider. Firstly, significant heterogeneity was observed across studies, which subgroup analysis attributed to differences in the studied population, participant gender, vitamin D status, LTL assessment methods, and the level of covariate adjustment. Factors such as BMI, study quality, and sample size did not contribute to this heterogeneity. Secondly, a remarkable publication bias was detected, potentially due to the search being limited to English-language articles, which might have excluded smaller studies published in other languages. Nevertheless, trim-and-fill analysis indicated that the impact of publication bias on the overall effect size was minimal. Third, the level of adjustment for potential confounding variables differed across studies, which might influence the observed associations. A more uniform approach to covariate adjustment could provide clearer insights. Lastly, all studies included in the meta-analysis were cross-sectional, which limits the ability to infer causality between 25(OH)D levels and LTL. Longitudinal studies are necessary for understanding temporal relationships.

In conclusion, this meta-analysis highlights a positive association between serum 25(OH)D levels and LTL in specific subgroups, including women, adults, and individuals with vitamin D deficiency. These findings suggest that vitamin D may play a role in maintaining TL in these populations. However, no significant associations were observed in men, children, or individuals with sufficient vitamin D levels, indicating that the relationship between vitamin D and LTL may vary based on demographic and health factors. Further research is needed to explore the underlying mechanisms and to confirm these findings in larger and more diverse populations.

## Author contributions

**Data curation:** Jie Shen, Lin Wang.

**Funding acquisition:** Jie Shen.

**Resources:** Jiyuan Liu, Zhichao Fan.

**Software:** Lin Wang, Zhichao Fan.

**Supervision:** Guanghui Li.

**Writing – original draft:** Jie Shen, Lin Wang, Jiyuan Liu, Zhichao Fan.

**Writing – review & editing:** Jie Shen, Guanghui Li.
